# Diagnostic and Vaccination Approaches for Newcastle Disease Virus in Poultry: The Current and Emerging Perspectives

**DOI:** 10.1155/2018/7278459

**Published:** 2018-08-05

**Authors:** Muhammad Bashir Bello, Khatijah Yusoff, Aini Ideris, Mohd Hair-Bejo, Ben P. H. Peeters, Abdul Rahman Omar

**Affiliations:** ^1^Laboratory of Vaccines and Immunotherapeutics, Institute of Bioscience, Universiti Putra Malaysia, 43400 Serdang, Selangor, Malaysia; ^2^Department of Veterinary Microbiology, Faculty of Veterinary Medicine, Usmanu Danfodiyo University, Sokoto, Nigeria; ^3^Department of Microbiology, Faculty of Biotechnology and Biomolecular Sciences, Universiti Putra Malaysia, Selangor, Malaysia; ^4^Department of Veterinary Clinical Services, Faculty of Veterinary Medicine, Universiti Putra Malaysia, Selangor, Malaysia; ^5^Department of Veterinary Pathology and Microbiology, Faculty of Veterinary Medicine, Universiti Putra Malaysia, Selangor, Malaysia; ^6^Department of Virology, Wageningen Bioveterinary Research, Lelystad, Netherlands

## Abstract

Newcastle disease (ND) is one of the most devastating diseases that considerably cripple the global poultry industry. Because of its enormous socioeconomic importance and potential to rapidly spread to naïve birds in the vicinity, ND is included among the list of avian diseases that must be notified to the OIE immediately upon recognition. Currently, virus isolation followed by its serological or molecular identification is regarded as the gold standard method of ND diagnosis. However, this method is generally slow and requires specialised laboratory with biosafety containment facilities, making it of little relevance under epidemic situations where rapid diagnosis is seriously needed. Thus, molecular based diagnostics have evolved to overcome some of these difficulties, but the extensive genetic diversity of the virus ensures that isolates with mutations at the primer/probe binding sites escape detection using these assays. This diagnostic dilemma leads to the emergence of cutting-edge technologies such as next-generation sequencing (NGS) which have so far proven to be promising in terms of rapid, sensitive, and accurate recognition of virulent Newcastle disease virus (NDV) isolates even in mixed infections. As regards disease control strategies, conventional ND vaccines have stood the test of time by demonstrating track record of protective efficacy in the last 60 years. However, these vaccines are unable to block the replication and shedding of most of the currently circulating phylogenetically divergent virulent NDV isolates. Hence, rationally designed vaccines targeting the prevailing genotypes, the so-called genotype-matched vaccines, are highly needed to overcome these vaccination related challenges. Among the recently evolving technologies for the development of genotype-matched vaccines, reverse genetics-based live attenuated vaccines obviously appeared to be the most promising candidates. In this review, a comprehensive description of the current and emerging trends in the detection, identification, and control of ND in poultry are provided. The strengths and weaknesses of each of those techniques are also emphasised.

## 1. Introduction 

Newcastle disease (ND) is one of the most important avian diseases that significantly affect poultry production all over the world [1]. From its first official report in 1926 at Newcastle Upon Tyne in England to date, ND has accounted for tremendous economic losses through numerous epidemics associated with high mortality, high morbidity, and many other production related losses. Consequently, the World Organisation for Animal Health (OIE) has included it among the list of diseases that require immediate notification upon recognition [2]. The aetiology of the disease is Newcastle disease virus (NDV), a negative stranded RNA virus whose 15.2 kb nonsegmented genome is organised into six genes encoding six structural proteins, namely, NP, P, M, F, HN, and L, as well as two nonstructural proteins, V and W [3]. Among these proteins, the F is generally considered to be a molecular marker of NDV virulence. According to the OIE, virulent NDV strains are diagnosed as those possessing multiple basic amino acid residues (arginine and lysine) at the F protein cleavage site located between amino acid positions 112-116, and a phenylalanine residue at position 117. On the other hand, isolates of low virulence are considered to be those with monobasic F cleavage site and a leucine residue at position 117 [2]. Unfortunately, this is not a universal rule of thumb, as some NDV strains adapted in wild birds such as pigeons and doves have been shown to be minimally pathogenic despite possessing the so-called polybasic F cleavage site [4, 5]. Furthermore, some of these pigeon paramyxoviruses have been reported to manifest a dramatic increase in virulence following a few passages in chicken, without the accompanying nucleotide changes in the entire F coding region [6, 7]. In addition, swapping the F genes of avirulent pigeon adapted NDV with that of a virulent NDV strain or vice versa did not lead to the change in the virulence of the generated chimeric viruses. These observations altogether signify that other factors independent of the F cleavage site are important in determining the virulence of NDV in chicken. Indeed, the HN and L proteins have recently been proven to be directly associated with NDV virulence [8, 9]. Hence, the biggest diagnostic dilemma is whether targeted evaluation of F cleavage site is still a reliable molecular indicator of NDV virulence or complete genome analysis is required to adequately predict the virulence potential of NDV isolates.

Epidemiological evidences indicate that NDV is constantly evolving, and its isolates are so far classified into more than eighteen phylogenetically distinct genotypes [14]. Regardless of their genetic variability however, all NDV isolates are serologically grouped into a single serotype called avian paramyxovirus type-1 [15]. This implies that immunity induced by any NDV strain should be able to cross-protect against challenge with any other virulent strain, because of the fairly similar antigenic properties shared by all NDV isolates. Indeed for over sixty years, both live attenuated and inactivated NDV vaccines have been extensively used to curb the economic menace of ND across the globe [16]. In particular, the live vaccines are known for their track record of high efficacy, due to their ability to efficiently replicate and induce a robust immune response following a single administration. They are also suitable for mass application via spray or drinking water [17]. Unfortunately, they are unable to block the replication of the heterologous virulent NDV despite protecting against overt clinical disease [18]. As a result, birds vaccinated with those vaccines may serve as reservoirs for virulent NDV, shedding a substantial amount of the infectious virus into the environment, leading to potential outbreaks among the nonprotected in-contact birds [19]. These shortcomings of the conventional vaccines collectively call for the need to improve the current vaccination strategies against the prevalent NDV strains across the globe.

Recently, the global poultry industry has witnessed an upsurge in the emergence of cutting-edge techniques for the identification and control of virulent NDV infection. These state-of-the-art technologies have no doubt demonstrated the potentials to overcome the loopholes of their conventional counterparts. Here, we comprehensively review the current and next-generation diagnostic and vaccination strategies for NDV, with special emphasis on the strengths and weaknesses of each of those techniques.

## 2. Etiology

### 2.1. Classification of NDV

Isolates of NDV are members of the genus* Avulavirus* in the family Paramyxoviridae. At present, a total of 15 avian paramyxovirus serotypes (APMV-1 to APMV-15) have been identified in different species of wild and domesticated birds, where they may be associated with respiratory disease and a drastic reduction in egg production [20]. Members of APMV-1 made up of NDV isolates are the most economically important and are genetically classified into two broad groups, class I and class II. Whereas members of class I isolates are grouped into only one genotype, isolates belonging to class II are further subdivided into genotypes I-XVIII which are all predicted to be virulent in chicken, except some isolates in genotypes I, II, and X [21, 22].

### 2.2. Molecular Biology of NDV

The genetic material of NDV is a negative stranded nonsegmented RNA that strictly adheres to the rule of six (genome size divisible by six), having a total size of 15186, 15192, or 15198 bp [23]. The genome houses six genes, namely, nucleocapsid protein (NP), phosphoprotein (P), matrix protein (M), fusion protein (F), hemagglutinin-neuraminidase protein (HN), and the large protein (L). Apart from the P gene which can be transcribed into three different mRNAs encoding one structural (P) and two nonstructural (V and W) proteins [24], all other viral genes are monocistronic encoding a single structural protein. The 3′ and 5′ ends of the viral genome constitute the leader and trailer regions, which accommodate the regulatory signals for virus transcription and replication [25].

**Figure 1:**
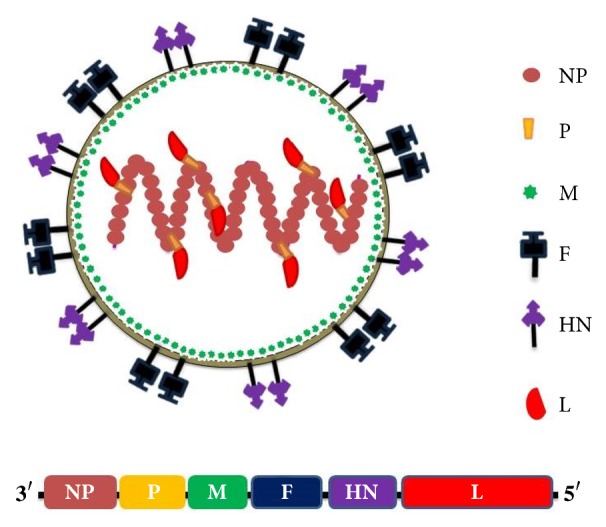
**Structural features of Newcastle disease virus**. A. Morphology of the virion showing the locations of the viral proteins. NP, P, and L proteins associate with the RNA genome to form RNP, while the M, F, and HN are membrane associated B. Arrangement of the genes in the viral genome.

Morphologically, NDV appears to be a pleomorphic enveloped particle with projections of F and HN spike glycoproteins (Figure 1) that participate in the initiation of virus infectious cycle (Sachin et al., 2011). Immediately beneath the viral envelope is the M protein, which is known to maintain the shape of the virus and assist in the packaging and release of the newly assembled viruses [26]. The other three proteins are intimately associated with the viral genome and are known to perform replication related functions. In particular, the NP precisely covers the entire viral RNA to form the ribonucleoprotein (RNP) which is the minimum template required for virus replication and mRNA biosynthesis [27]. The L protein is the RNA dependent RNA polymerase that serves as the viral replicase and transcriptase during the infectious cycle, while the P protein is the cofactor of the polymerase [8].

### 2.3. Epidemiology and Emergence of the Virulent NDV

**Figure 2:**
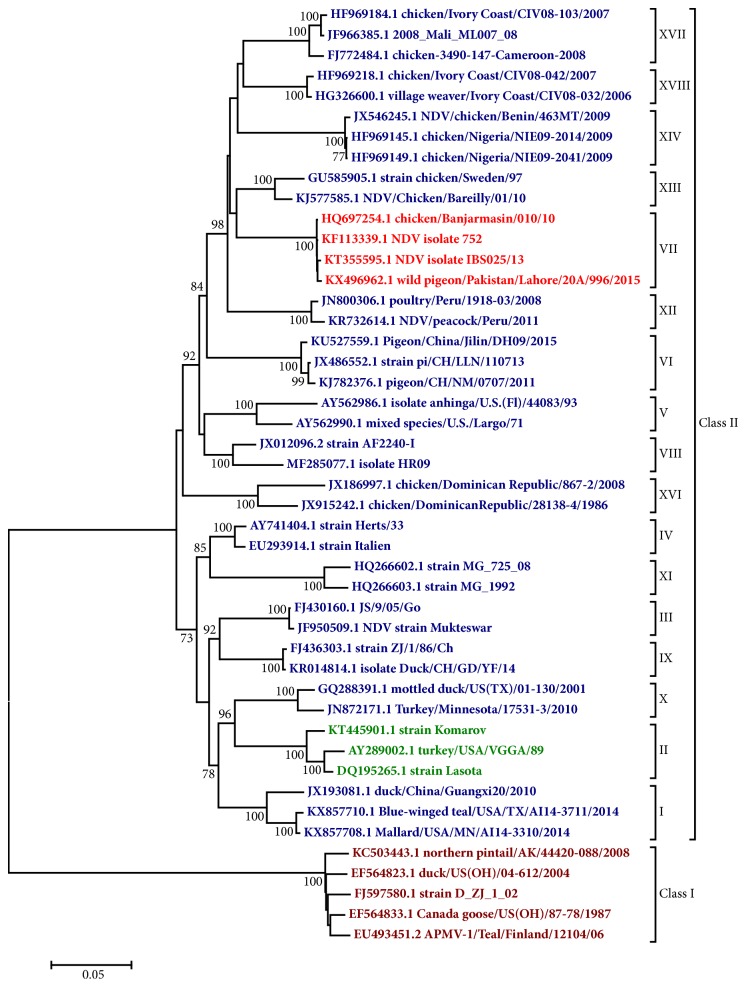
**Phylogenetic relationships of Newcastle disease virus genotypes (shown in roman numbers) using the complete F gene coding sequences (1662bp).** Red coloured taxon represents the group currently causing the wave of the fourth ND pandemic. Taxon containing the current vaccine strains is shown with green colour. The evolutionary history was inferred using the Neighbor-Joining method [10]. The optimal tree with the sum of branch length = 1.60126925 is shown. The percentage of replicate trees in which the associated taxa clustered together in the bootstrap test (1000 replicates) is shown next to the branches [11]. The tree is drawn to scale, with branch lengths in the same units as those of the evolutionary distances used to infer the phylogenetic tree. The evolutionary distances were computed using the Maximum Composite Likelihood method [12] and are in the units of the number of base substitutions per site. The analysis involved 46 nucleotide sequences. Codon positions included were 1st+2nd+3rd+noncoding. All positions containing gaps and missing data were eliminated. There were a total of 1656 positions in the final dataset. Evolutionary analyses were conducted in MEGA6 [13].

In the last nine decades, four major economically devastating NDV panzootics have occurred. The first one, which began simultaneously in Asia and Europe around the mid-1920s, was spreading to the rest of the world rather slowly and took about twenty years to become fully established [28]. The second pandemic however became full pledged within four years [29] presumably due to the increased commercialisation of poultry industry globally, as well as the enhanced international trade of captive cage birds, which were shown to be the reservoirs of virulent NDV in different parts of the world [30, 31]. The third panzootic believed to be caused by genotype VI isolates in the mid-1980s occurred among the racing pigeons but eventually affected several bird species and became difficult to control due to the relative lack of absolute control in the racing pigeon husbandry [32]. Finally, the fourth pandemic which is currently ongoing, is believed to have started from the late 1980s and has been linked to several economic losses in a vast number of countries across the South East Asia, Middle East, Europe, Africa, and America [33–35]. This particular panzootic has been shown to be caused by the genotype VII group of NDV which currently constitute the most rapidly evolving strains of the virus (Figure 2). Given the recent expansion in the geographic distribution and host range of some of these genotype VII strains, the fifth panzootic is strongly anticipated in the near future [36].

The emergence of virulent NDV isolates is certainly a serious concern in the global poultry industry. Apparently, lentogenic strains predominantly harboured by wild birds [37] are among the major reservoirs for the emergence of the virulent NDV in poultry. Through ecological contact interfaces, these lentogenic viruses have been shown to be easily be transmitted from wild birds to domesticated poultry, where they are silently maintained without causing any clinical disease [38]. However, continuous multiplication of lentogenic NDV in chicken is a potential risk factor for the emergence of the virulent NDV. Meng et al. [39] serially passaged a duck origin lentogenic NDV strain 10 times in chicken air sacs and showed that the virus dramatically acquired virulence, as measured by standard pathogenicity assessment indices. When ultra-deep sequencing of the partial genome encompassing the F cleavage site of the passaged virus was performed, it was revealed that the proportion of the virulent NDV variants within the pool of the viral quasispecies gradually accumulated as the passage number increased [39]. Similarly, vaccine derived NDV strains used in domesticated chicken have been reported to occur in wild birds [37], which further provides evidence of virus exchange between the wild and domesticated avian species at ecological contact points. Thus, the continuous circulation and maintenance of lentogenic NDV between the wild and domesticated birds constitute a huge threat for the emergence of virulent NDV strains of huge economic consequences.

### 2.4. Pathotypes and Pathotyping of NDV

All virulent NDV isolates require immediate notification to the OIE [40]. Hence, pathotype identification is necessary for a complete diagnosis of NDV in poultry. Using molecular based assays, the predicted amino acid sequence of the F cleavage site can be used to classify NDV isolates into virulent and avirulent strains [41]. Isolates with polybasic F cleavage site and phenyl alanine at position 117 are considered virulent, while those with monobasic F cleavage site and leucine residue at position 117 are classified as avirulent. Based on some* in vivo* pathogenicity assessment tests, NDV isolates can also be classified into velogenic (very virulent), mesogenic (moderately virulent), and lentogenic (avirulent) pathotypes [42]. The commonest of such tests is the mean death time (MDT) performed in 9-10-day-old embryonated chicken eggs, which is the average time (in hours) taken for the mean lethal inoculum of the virus to kill all the inoculated embryonated eggs [43]. As a general rule, isolates are diagnosed as velogenic when their MDT is 40-60 hours or mesogenic if they have MDT values of 60-90 hours. Lentogenic strains normally have MDT values of greater than 90 hours [44]. At present, the most widespread NDV pathotyping tool is the intracerebral pathogenicity index (ICPI) performed in 1-day-old SPF chicks [45]. It is normally scored between 0 and 2, with virulent strains having values from 1.3 to 2.0, while the values for mesogenic strains range from 0.7 to 1.3. Lentogenic isolates generally have ICPI values of 0.0-0.7 [46]. Finally, a useful but less popular virulence determination test is the intravenous pathogenicity index (IVPI) performed in 4-6-week-old SPF chicken. Its scores range from 0 to 3 and it is normally proportional to the virulence of the virus [46]. Thus, isolates with IVPI value of 0.0 are lentogenic, while mesogenic strains have values between 0.0 and 0.5, and the values for velogenic strains range between 0.5 and 3.0. In summary, for an isolate to qualify as notifiable to the OIE, it has to be classified as virulent by possessing at least one of the following: poly basic F cleavage site, MDT value of 40-60 hours, and ICPI value of > 1.3 or IVPI > 0.5.

## 3. Diagnosis

### 3.1. Diagnostic Dilemma

Poultry respiratory pathogens such as avian influenza, infectious bronchitis, and infectious laryngotracheitis viruses are all considered differential diagnoses that can easily be confused with NDV based on their clinical presentation [47]. In fact, some avian paramyxoviruses such APMV-3 and 7 may even cross react with NDV in routine serological diagnosis [48]. Thus, it is important to rapidly identify the strains of NDV and differentiate them from other closely related pathogens so that correct intervention for disease control can be applied. As a pathogen of several avian hosts, NDV demonstrates a wide spectrum of virulence that ranges from highly fatal to subclinical disease. This virulence spectrum is largely controlled by the genetic makeup of both the virus and avian hosts. Wild birds for instance can maintain highly virulent NDV without showing obvious clinical disease symptoms [49]. Backyard chicken, geese, and ducks which are naturally less susceptible to virulent NDV are also incriminated in maintaining the virus in the environment [50]. Furthermore, exotic pet birds kept in close proximity to commercial poultry have been shown to harbour virulent NDV strains that are genetically highly related to those obtained in the commercial farms [51]. This occurrence of virulent NDV in apparently healthy wild and backyard poultry not only constitutes a huge diagnostic challenge but also represents a big biosecurity threat to the commercial poultry industry.

The OIE recommended* in vivo* tests used to identify virulent NDV isolates have certainly proven to be useful in ND diagnosis. However, they often give contradictory results. An isolate classified as mesogenic using the MDT may turn out to be velogenic based on the ICPI or IVPI tests [52]. Furthermore, pathotyping of NDV isolates obtained from species other than chicken may not yield very accurate results until the isolates are passaged in chicken or chicken embryonated eggs. More so, the ICPI test regarded as the most robust OIE recommended pathogenicity test may not perfectly depict the real virulence of the virus because it utilises a route that does not represent the natural route of NDV infection [6, 7]. These diagnostic dilemma leads to the conclusion that the best indicator of NDV virulence in a particular avian species should be the experimental infection of a statistically significant number (≥10) of young and adult birds with a standard amount of the virus inoculum via natural routes [53]. There is therefore the need to improve the current pathotyping systems so that virulent NDV can be rapidly and accurately identified and contained before devastating loss of poultry is incurred.

An important virus related factor that contributes to the dilemma in the diagnosis of virulent NDV is the amino acid composition of the F cleavage site. According to the OIE, virulent NDV isolates are identified by the possession of multiple basic amino acids at the F cleavage site, which can be cleaved by all furin-like intracellular proteases ubiquitously distributed all over the body [2]. On the other hand, avirulent isolates are those possessing monobasic F cleavage site cleavable by extracellular trypsin like proteases found largely in the gastrointestinal and respiratory systems. This makes the molecular prediction of the virulence potential of NDV to be solely based on the chemistry of F cleavage site [54]. Contrastingly, emerging evidences indicate that other NDV genes might considerably contribute to the viral virulence. In one study, a single passage of a recombinant NDV strain LaSota encoding a velogenic F cleavage site in pigeon leads to a dramatic increase in ICPI from 1.3 to 1.7 without any change in the entire nucleotide sequence of the F gene [55]. In another study, pigeon derived NDV strains expressing the velogenic F cleavage site were shown to be completely avirulent in chicken especially at first passage. However, after several passages, the viruses became highly virulent even though no obvious nucleotide substitution in the entire F gene of the virus was observed [56]. This indicates that virulence of NDV is multigenic and that other factors independent of the F cleavage site are crucial in determining the virulence of the virus. Thus, the dilemma in the diagnosis of virulent NDV demands a revisit and improvement of the current pathotyping tools so that the virulence of NDV isolates can be more accurately predicted.

### 3.2. Clinical Diagnosis

#### 3.2.1. Clinicopathological Features

On the basis of the clinical and pathologic manifestations, five different forms of ND are recognised [57]. The severest form is the velogenic viscerotrophic ND (VVND) characterised by mortality and morbidity rates approaching 100% [58]. It is associated with conjunctivitis, nasal discharges, dyspnoea, diarrhoea, ruffled feathers, prostration, tremors, and paralysis. At postmortem, ulcerative haemorrhages may be observed throughout the digestive tract, especially at the proventriculus-gizzard junction and in the caecal tonsils [42]. Necrotic foci may also be also observed in some internal organs such as the spleen, liver, and gut associated lymphoid tissue (GALT). Histologically, the spleen and the Peyer's patches show microscopic evidence of necrosis and haemorrhage. In the nervous system, apart from perivascular cuffing, no neurological lesions due to VVND are observed even among birds that died showing neurological symptoms [53].

Another form of the disease is velogenic neutrotropic ND (VNND) characterised by neurological and some respiratory clinical signs with no gastrointestinal involvement. Typically, the affected birds manifest opisthotonus, tremors, head twisting, and paralysis. Gross lesions are often absent even among birds that died showing typical symptoms. However, at histology, necrosis of Purkinje fibres as well as perivascular cuffing are highly encountered [59]. Mesogenic ND (MND) is also associated with neurological and respiratory symptoms with a very low mortality rate. Its clinical signs under field conditions are those associated with drop in egg production and mild to moderate respiratory illness [48]. Gross pathological findings are also minimal, involving only a slight splenomegaly and other lesions as a result of secondary bacterial infections. Histopathological findings include gliosis and perivascular cuffing which may or may not be accompanied by pancreatic necrosis [42].

The other forms of the disease are the lentogenic ND (LND) and asymptomatic enteric ND (AEND) which are generally associated with mild or no evidence of clinical disease. In fact, the mild respiratory disease associated with the LND is only in young but not in adult chicken. Experimental infection to study the pathology of lentogenic B1 and Q4 strains in 4-week-old chicken produced no apparent clinical signs [60]. Postmortem findings may be absent or at best may involve mild hemorrhages in the tracheal and pulmonary tissues. At histology, lymphoid follicles proliferation in the tracheal tissue might be encountered. There may also be the loss of cilia, infiltration of lymphocytes, and squamous cell metaplasia [61]. Finally, the AEND is completely avirulent, causing only the replication of the virus in the intestinal tissues of the infected chicken.

#### 3.2.2. Differential Diagnosis

The clinicopathologic picture of ND gives important clues in making clinical diagnosis. However, a number of viral and bacterial diseases may manifest similar clinical features that could be confused with ND. The commonest differentials of ND include highly pathogenic avian influenza, infectious bronchitis, infectious laryngotracheitis, and diphtheritic form of fowl pox. Others include fowl cholera, mycoplasmosis, and psittacosis in psittacine avian species [62]. Distinguishing ND from all these diseases is a crucial task in arriving at tentative diagnosis.

### 3.3. Virus Isolation

This is regarded as the gold standard method for the definitive diagnosis of ND and is often used for validating the results from other detection methods [63]. The choice of samples required for virus isolation is determined by the sites of virus replication and routes of viral shedding. In live birds, samples required include the cloacal and oropharyngeal swabs collected in isotonic solution with or without antibiotics. If the birds are already moribund or have recently died, samples should include lungs, kidney, liver, intestine, spleen, and caecal tonsils collected separately or as a pool, in addition to the cloacal and oronasal swabs [2]. To isolate NDV, processed samples are primarily inoculated into the allantoic cavity of 9-10-day-old specific antibody free chicken embryonated eggs. After about 4-7 days of incubation, hemagglutination test (HA) is used to detect the presence of the virus in the infected allantoic fluid. However, since other viruses such as avian influenza and APMVs might also possess HA activity, it is always necessary to further confirm the identity of the virus using other diagnostic tests such as hemagglutination inhibition test (HI) using NDV specific antisera or molecular tests. It should be emphasised that some serological cross-reactivity might occur between NDV and APMV-3 or APMV-7 [63]. This can however be circumvented by the use of a panel of monoclonal antibodies specific for NDV.

Isolation of NDV can also be performed in primary cell cultures such as chicken embryo fibroblasts (CEF), DF-1, chicken embryo kidney (CEK), chicken embryo liver (CEL) cells, and avian myeloblasts (QM5) which are all highly permissive to the virus [64]. The cells are infected with clinical samples and monitored for cytopathic effects (CPE) such as cell rounding, syncytia formation, and cell death [65]. Importantly, isolation of avirulent NDV strains in cell culture might require the addition of exogenous trypsin, since their monobasic F cleavage site can only be activated by extracellular trypsin like proteases. Worthy of note, some pigeon adapted NDV strains (PPMV-1) can only be isolated via cell culture but not using embryonated eggs [6, 7]. Whenever such viruses are suspected, it is advisable to attempt virus isolation in both embryonated eggs and cell culture. Generally, the use of cells in NDV isolation gives a lower yield of the virus. Hence, even after virus isolation in cells, it might be necessary to propagate the isolated virus in embryonated eggs if the downstream application requires the use of the virus in large quantities [32].

### 3.4. Serological Diagnosis

The diagnostic relevance of serology in NDV surveillance is considerably belittled by its inability to differentiate vaccinated from infected animals (DIVA). Nevertheless, serological tests remain a valuable tool used by many diagnostic laboratories to assess the humoral immune responses following vaccination [66]. The simplest and most inexpensive serological test for NDV is HI which measures the ability of NDV specific antibodies to inhibit the agglutination of RBCs by the NDV particles. The test is normally performed using standard amount of NDV (4 or 8 HAU) as HA antigen [67]. The reciprocal of the highest serum dilution that completely blocks agglutination is the HI titre. In birds whose HI titres are monitored closely (such as vaccinated birds), sudden rise in the titre might be indicative of exposure to field NDV strain, even though APMV-3 has also been reported to cause same [68].

Another robust test used in NDV serology is ELISA. In the last few years, several ELISA kits based on whole or part of the virus antigen have been developed for rapid diagnosis of ND [69, 70]. Many of these kits are commercially available in the form of sandwich, competitive or indirect ELISA [44]. They are highly sensitive and produce results that pretty well correlate with HI test results. Whereas in the HI tests, only the antibodies directed against the HN protein are detected, ELISA platforms utilising whole virus as antigens can potentially detect antibodies directed against all the proteins in the NDV particle. With the growing interests in subunit ND vaccines for disease control [71, 72], it is possible to develop ELISA tests that can differentiate antibodies due to vaccination from those due to infection. Makkay et al. [69] developed antibody detection ELISA using recombinant NP protein expressed in insect cells as antigen and demonstrated the powerful DIVA property of the test. Similarly, Zhao et al. [73] demonstrated that ELISA based on recombinant full length NP expressed in bacterial cells was able to detect NDV antibodies with high sensitivity in sera obtained from vaccinated birds even though some levels of cross-reactivity with antibodies raised against other APMVs were observed. Interestingly, the cross-reactivity was completely eliminated when only the C terminal extension of the NP was used as a diagnostic antigen [73]. Certain limitations however make these tests less routinely used compared to the HA/HI tests. Apart from being expensive and unsuitable in the field, these monoclonal antibody (Mab) based ELISAs may not be able to detect certain strains of NDV that might have some mutation in the single epitope against which the monoclonal antibody was raised. Nevertheless, they still remain good diagnostic tests for ND surveillance.

Virus neutralisation test (VNT) is yet another powerful serological test particularly useful in measuring NDV specific neutralising antibodies. The test is performed by mixing a serially diluted serum with a standard amount of NDV (for instance 100 PFU) followed by infection of the cultured DF-1 cells with the virus-serum mixtures [74]. NDV specific neutralising antibody titre is determined by the highest serum dilution that demonstrated a clear CPE in the cultured DF-1 cells after about four days of incubation. The test is the most superior tool for assessing neutralising antibody titre following vaccination. However, it is highly laborious and very slow, yielding results only after nearly one week. Interestingly, Chumbe et al. [75] reported the development of an improved VNT using a recombinant NDV engineered to constitutively express GFP. The newly developed assay has been shown to give conclusive results within 24 hours without the need for any additional staining procedure. Furthermore, its correlation with conventional VNT is much higher than how HI or ELISA is correlated with the conventional VNT. The test is therefore an emerging rapid method of quantifying neutralising antibody titre. However, it will be better suited in the assessment of vaccine protective immunity than in NDV surveillance.

### 3.5. Molecular Based Assays

#### 3.5.1. Reverse Transcription-Polymerase Chain Reaction (RT-PCR)

The shortcomings of the conventional diagnostic techniques warrant the need for the development of more rapid, yet very accurate methods of ND diagnosis in poultry. The most commonly used molecular test in NDV diagnosis especially in the developing countries is RT-PCR. The test can rapidly and accurately detect viral genome in clinical samples with high sensitivity especially if appropriate samples are taken. It is usually designed to simultaneously detect and identify the pathotype of the virus [76] by targeting the F gene portion encompassing the F cleavage site, followed by restriction fragment length polymorphism using* Bgl*I whose digestion pattern classifies NDV isolates into lentogenic, mesogenic, and velogenic strains [77]. Nowadays, molecular NDV pathotyping is predominantly based on RT-PCR followed by the analysis of the putative amino acid composition of the F cleavage site [78]. Molecular based pathotyping is therefore a good alternative to the conventional virus isolation technique which in addition to being slow might also require specialised containment facilities [79]. However, given the continuous emergence and evolution of NDV [80], there is a need to regularly update the primers used in the assay so as to account for the variants that might escape detection as a result of mutation in the primer binding site.

#### 3.5.2. Quantitative Polymerase Chain Reaction (qPCR)

The qPCR assay is not only faster and less cumbersome than the conventional diagnostic techniques, but also provides equal or even greater sensitivity of virus detection than the gold standard virus isolation method. In many countries including the United States, matrix gene and fusion gene based qPCR assays are often used as standard methods for NDV screening and pathotyping directly from clinical samples [81]. The choice of matrix gene detection in NDV screening is informed by its highly conserved nature in the NDV genome. Thus, matrix gene assay is able to detect most of NDV isolates especially those that belong to class II [49]. Because most of the class I isolates often escape detection using this assay, an improved assay called matrix-polymerase multiplex qPCR was developed by utilising a conserved region on class I L gene for primer and probe design. This new assay not only detected the previously undetectable NDV isolates, but also works in conjunction with the matrix gene assay under the same experimental settings [5]. Matrix gene assay is therefore a rapid test for NDV screening in many countries.

For NDV pathotyping, an F gene based qPCR assay that differentiates the low virulence viruses from the virulent NDV strains was developed [82, 83]. Although this assay was designed to detect NDV isolates in the United States, it was found to be useful in detecting most isolates around the world except a few ones that possess nucleotide substitutions at the probe binding sites [84]. Further investigation revealed that some of the isolates that escaped detection using this primer and probe combination had at their F cleavage site, a lysine (K) residue at position 114 instead of the conventional glutamine (Q) at that position. Indeed a number of other nucleotide differences were observed between the genome of those viruses and the F gene probe used in this assay. Interestingly, when a new probe that took the above nucleotide differences into consideration was designed and tested, isolates that initially escaped detection using the earlier F gene assay were all identified with this improved platform [4]. In our laboratory, we recently developed a highly sensitive probe based qPCR system for the identification of virulent NDV strains circulating in Malaysia [18]. Although the qPCR assay is currently validated for NDV screening from clinical samples, it is imperative to continuously monitor the genetic diversity of the evolving NDV isolates so that the primers and probes can periodically be updated to identify all the possible escape mutants. Indeed, a natural recombinant strain NDV IBS025/13 isolated recently in our laboratory was shown to escape detection using our established primers and probes, due to a two-nucleotide substitution at the extreme 3′ end of the probe binding site [85]. When the probe's sequence was updated, all the virulent NDV strains were successfully detected.

In addition to disease identification and pathotyping, the qPCR assay can also be used in the quantification of viral load in different organs [86] or virus shedding from the vaccinated animals following challenge with the virulent NDV strain [18, 87]. The traditional methods of virus shedding assay are the end point dilutions such as mean tissue culture infective dose (TCID_50_) and median egg infective dose (EID_50_) or plaque assay [88]. These methods are very laborious, requiring a large number of eggs or multiple plates of seeded cells for them to be fully accomplished. It also takes several days for these assays to be completed. Hence, emerging trends in this regard include the use of qPCR systems which are highly sensitive, specific, and reliable in the detection and quantification of virus in clinical samples within just a couple of hours.

#### 3.5.3. Non-PCR Amplification Techniques

Recently, a simple, sensitive, and inexpensive diagnostic assay called loop mediated isothermal amplification (LAMP) test was developed for the rapid detection of the genetic materials of infectious agents. The principle of the assay is a strand displacement reaction that forms a stem loop structure, allowing sensitive and specific amplification of the target template [89]. The specificity of LAMP test is due to its ability to detect six independent regions during the amplification reaction [90, 91]. The assay is performed under isothermal conditions and only requires 4-6 primers, a DNA polymerase, and a water bath [92]. It is therefore highly applicable to diagnostic laboratories in the developing countries where more sophisticated instruments are not readily available. In addition, the LAMP amplified product can easily be detected with naked eyes by a simple colour change. To facilitate results visualisation, SYBR green is traditionally added to the LAMP mixture prior to the isothermal incubation [93]. Pham et al. [94] developed a LAMP based assay for the detection of NDV directly from clinical samples and showed that its sensitivity and specificity are similar to those of nested PCR, yet it is simpler and inexpensive. Similarly, Kirunda et al. [95] reported the use of RT-LAMP to detect NDV RNA from cloacal and tracheal swabs obtained from chicken in less than one hour. Thus, the inexpensiveness, rapidity, specificity, and sensitivity of the LAMP assay have made it highly useful in ND diagnosis especially in the rural areas. However, the difficulties in the design of the four primers that independently target different sites on the template, as well as the challenges in LAMP multiplexing, are some of the limitations of this assay.

### 3.6. Microarray Hybridisation Techniques

Molecular based diagnostics have no doubt revolutionised the world of infectious disease diagnosis by simultaneously being simple, rapid, and sensitive in identifying disease agents. However, most of these assays may only detect a single or few organisms at a time. Microarrays have the potential to concurrently monitor, detect, and characterise hundreds to thousands of targets without compromising the assay's sensitivity and specificity [96]. They are essentially made up of solid supports equipped with several DNA probes of known identities capable of hybridising to their targets in clinical samples. Thus, they are said to be efficient platforms for pathotyping, genotyping and disease biomarkers identification. A lot of DNA microarray systems have been developed for typing pathogenic agents [97, 98]. Using these microarray hybridisation systems, Lung et al. [99] reported the typing of NDV with a detection limit of as low as 10^1^–10^3^  TCID_50_/ml. Indeed, NDV and avian influenza virus were simultaneously detected using this technique [99], demonstrating the potential of DNA microarrays in the detection of mixed infection. Different NDV pathotypes as well as the H5 and H7 avian influenza viruses (AIV) were also detected simultaneously using a DNA microarray hybridisation system developed by Wang et al. [100]. More recently, newly developed microarray diagnostics including the multiplex luminex suspension microarray systems were used for the simultaneous detection of NDV, AIV, infectious bursal disease virus, and infectious bronchitis virus in either single or mixed infections [101, 102]. Therefore, it will not be out of place to assert that DNA microarrays are among the emerging diagnostics that in the near future could outperform the conventional NDV typing techniques, given their sensitivity, specificity and the ability to simultaneously detect multiple pathogens/pathotype in clinical samples.

### 3.7. Biosensor Diagnostics

Biosensors are analytical systems made up of biorecognition molecules and physicochemical detectors called transducers, capable of converting biomolecular interactions into measurable signals [103]. They provide an incredibly sensitive and inexpensive platform for the rapid detection and identification of infectious disease agents [104]. Label-free biosensors which provide a means of continuously monitoring in real time, the binding affinity, and kinetics of biomolecules over time [105] are among the emerging diagnostic tools in the 21^st^ century. Typical example is the surface plasmon resonance (SPR) which directly quantifies the biomolecular interaction on its immobilised gold surface via the detection of changes in refractive index. Using this SPR technology, a number of bacteria [106, 107], viruses [108, 109], and other infectious agents [110] have been rapidly and selectively diagnosed. Recently, a label-free immunosensing system using excessively tilted fibre grating coated with gold nanospheres was developed. The system is highly sensitive, capable of detecting as little as 5 pg of NDV [111]. It is thus more sensitive than the qPCR assay whose detection limit is around 10 pg of the virus. Furthermore, this immunosensing method has a short detection time and does not require any sophisticated equipment. Therefore, considering their advantages over other diagnostic technologies for NDV, it is envisaged that the clinical application of biosensors will improve in the nearest future. So far, translating this biosensor technology from the detection in laboratory solutions to direct clinical samples remains a major challenge, perhaps due to the complex matrices that characterise the clinical samples [104]. Notably, most biosensors are highly sensitive to environmental factors such as temperature and pH. Nevertheless, several approaches are being developed to overcome those sample matrices effects.

### 3.8. Next-Generation Sequencing

Next-generation sequencing (NGS) is one of the most recent tools that have revolutionised the diagnosis of infectious diseases [112]. It is not only important in tracking disease epidemics, it also facilities the rapid, sensitive, and specific detection and differentiation of mixed infections within a single host [113]. It can also be used to detect low frequency variants which would otherwise escape detection using the common diagnostic tools. In addition, it is currently the most throughput tool used in the discovery of novel viruses associated with unknown diseases [112]. Hence, its application in the development of advanced diagnostics cannot be over emphasised. Although different platforms of NGS are constantly emerging, they all technically involve three major steps: sample preparation, sequencing, and data analysis [114]. Variations exist largely in the sequencing techniques. Currently, most NGS platforms aimed at viral diagnosis are focusing on improved sequence reads and speed of the assay. Recently, NGS based characterisation of genotype VI NDV in the United States has been reported to reveal a previously unknown genetic diversity of the virus with evidence of its continuous evolution [115]. Satharasinghe* et al*. [85] also discovered a naturally occurring hybrid NDV in Malaysia using NGS techniques with over 99% coverage. In addition, NGS has recently been applied to simultaneously characterise the genomic sequences of multiple avian paramyxoviruses [116]. More so, differentiation of virulent from avirulent strains of class II NDV was successfully and rapidly achieved using NGS based on pyrosequencing procedure [117]. These features altogether make NGS the most promising emerging technique for NDV discovery and diagnosis. So far, the only disadvantage of the assay is its perceived high cost of sample run. However, with the increasingly cheaper sequencing services, NGS tools are likely to become routinely available for viral diagnosis given their speed, accuracy, and multiplexing ability.

### 3.9. Random Priming Technologies

Identification of new viruses using the conventional RT-PCR or even qPCR is solely dependent on the assumption that the unknown virus is somehow identical to the previously sequenced viruses at least around the so-called conserved regions. However, this is not always the case owing to the high genetic diversity and evolution of RNA viruses [14, 118]. Consequently, periodical modification of the existing assays is necessary to account for mutants that could escape detection using these molecular based assays. Furthermore, in the case of mixed infection, these assays will only amplify genomes that more closely match the primers and probes and not necessary the most abundant in the pool of the samples [81]. Therefore, in order to overcome these challenges, it is necessary to develop more reliable and sensitive assays that are more robust than the existing ones.

Random priming techniques such as sequence independent single primer amplification (SISPA) have the potential to overcome these shortcomings. They work on the principle of random amplification of the genomic RNA, sequencing, assembly, and analysis of the sequence reads [119]. Using this SISPA method, full genome coverage of several viruses has been achieved within a short time and at a cost effective rate [120, 121]. However, for effective results, it is always essential that the samples contain large number of virus particles. In addition, pretreatment of the samples with DNAse-I may reduce the contamination from the host genome [119]. Interestingly, with the availability of next-generation sequencing (NGS) platforms that allow the detection of low frequency variants in a pool of samples [122], SISPA technology can be greatly enhanced. Indeed, Chrzastek et al. [123] reported the use of SISPA in conjunction with NGS to identify NDV in a mixture of viruses where its concentration is as low as 3log⁡10  EID_50_. As a matter of fact, complete genome sequence of NDV was obtained from samples containing mixed viruses where the titre of NDV was only 4.5log⁡10  EID_50_ [123]. Thus, rapid and accurate identification of NDV can be achieved using this system especially under outbreak situation where thousands of birds might be at risk of dying due to the disease. At present, the use of this assay is limited to virus discovery and other metagenomics applications because of its high cost per run. However, with the increased advancement in sequencing technologies, the assay is expected to be much cheaper in the nearest future and more available as a routine clinical diagnostic test.

## 4. Vaccination Strategies

Due to their role in minimising the traffic of pathogenic organisms in and out of the poultry facilities, tight biosecurity barriers are indispensable in the control of avian diseases [124]. However, even with a good biosecurity practice, vaccination is still required to optimally protect the birds against the economically devastating diseases such as ND. Hence, effective poultry disease control program demands a combination of strict biosecurity measures with vaccination regimen. The strategies for ND vaccination are broadly classified into two: the conventional methods developed in the 1940s as well as the recently emerged methods based on recombinant DNA technology. Here, a concise description of these methods is provided, with particular reference to strengths and weaknesses of each method.

### 4.1. Conventional Vaccines

#### 4.1.1. Live Attenuated Vaccines

The isolation of naturally occurring highly immunogenic NDV strains of low or intermediate virulence in the last 7 decades has certainly ameliorated the economic losses due to ND in many countries [125, 126]. To date, a number of lentogenic NDV strains such as B1, F, LaSota, V4, and I_2_ are extensively used as live vaccines for disease control [44]. Among these vaccine strains, LaSota is obviously the most widely used in different countries because of its superior immunogenicity. B1-based live ND vaccines may not be as immunogenic as LaSota but are renowned for their highly attenuated feature with no postvaccinal respiratory reactions in birds. In the case of V4 and I_2_, their major selling point is thermostability; they can tolerate elevated temperatures in the absence of cold chain, making them especially valuable in villages with limited refrigeration capabilities [127]. Other NDV strains used as popular live vaccines include the Komorov and Mukteswar strains, both of which are mesogenic and therefore suitable as booster vaccines following priming with lentogenic isolates [128].

All live attenuated ND vaccines are known to stimulate both mucosal and systemic immune responses similar to those of the natural infection, because of their ability to replicate in chicken irrespective of the site of administration [129]. Furthermore, a single administration of 10^5^  EID_50_ of live NDV vaccine is enough to rapidly stimulate an immune response capable of inducing a 100% protection against clinical disease, even though the shedding of the challenged virulent virus via the cloacal and oropharyngeal routes may still occur. In fact, there are indications that the replication and shedding of the virulent virus can be substantially reduced when much higher doses of the live vaccines are administered [21, 22]. As this is not an economically viable option because of the high cost of vaccination per bird, improved and cost effective vaccination approaches are required to tackle the problem of virus shedding associated with the conventional ND vaccines. An important factor that determines the effectiveness of vaccination is the tissue tropism of the vaccine. Conventional live attenuated NDV vaccines including the famous LaSota strains are predominantly respirotropic, inducing stronger mucosal immunity in the respiratory airways where initial exposure to the virus might occur. Other vaccines such as the VGGA strains are more of enterotropic [130], stimulating gut mucosal immunity among the vaccinated birds. Interestingly, we isolated a natural recombinant virulent NDV strain IBS025/13 [85] which demonstrates a strong tissue tropism in both the respiratory and gastrointestinal systems. It is our belief that this dual tropism feature of the virus and its high yield in chicken embryonated eggs make it a good candidate for future development of live attenuated vaccines against ND in chickens.

Perhaps the greatest advantage of live NDV vaccines is their suitability for mass application via drinking water or spray, making their total cost from the point of production to administration highly inexpensive [131]. In addition, the vaccine virus from the vaccinated birds may spread to the suboptimally vaccinated ones in the vicinity, thereby contributing to the overall herd immunity [132]. Furthermore, some of these attenuated strains such as NDV strain I_2_ have been shown to be naturally thermostable [127], requiring little or no cold chain maintenance, making them suitable vaccine candidates in the rural areas where electricity supply can be grossly inadequate. Nevertheless, in spite of all the benefits of these vaccines, they have their shortcomings. First and foremost, the live viruses may possess the potential to revert back to virulence and cause clinical disease in the vaccinated birds. Secondly, depending on the vaccine strain used, these vaccines may induce postvaccination respiratory reactions in young birds which, if severe, could predispose the birds to secondary bacterial infections [133]. In addition, the vaccines are largely based on genotypes I or II strains which are phylogenetically divergent from the currently prevalent genotypes in different countries. Hence, although the vaccines are still protective against clinical signs and mortality caused by any NDV isolate, their inability to block virus shedding postchallenge ensures the continuous presence of the virulent virus in the environment. This is particularly more dangerous with genotype VII isolates, whose shedding from LaSota vaccinated birds is significantly higher than those of other genotypes [18]. Therefore, considering the above limitations, the live attenuated vaccines must be used with utmost care and that the state-of-the-art vaccines are urgently needed to address these weaknesses of the conventional live attenuated vaccines.

#### 4.1.2. Inactivated Vaccines

Immunisation of chicken with inactivated vaccines is the earliest strategy for ND control. The vaccines are produced by growing any NDV strain of interest to high titres followed by its inactivation using physical or chemical methods [134]. Since the viral surface glycoproteins (F and HN) are the most important determinants of neutralising antibodies, methods used in viral inactivation should be those that spare the immunogenic epitopes of those proteins. Among the chemicals used for the inactivation, binary ethylenimine (BEI) and formaldehyde are the most popular [135]. The vaccines are normally prepared in emulsions of mineral oil (water-in-oil) and administered intramuscularly or subcutaneously. It is generally required that the ratio of water phase to oil phase strikes a balance between the vaccine stability and its viscosity, such that the vaccine remains stable and yet not difficult to administer due to high viscosity of the emulsion [136]. Because these vaccines cannot replicate and spread horizontally among the vaccinated birds, they are not suitable for mass application. Rather they are administered individually preferably via the parenteral route, making the entire process hectic and expensive. The same nonreplicating feature however makes them safe with no risk of reversion back to virulence [137]. Therefore, it is imperative to optimise the conditions for developing these vaccines as “too much inactivation” may destroy the immunogenic epitopes and mild exposure to the chemical or irradiation may not be sufficient to inactivate the virus [135]. For best results, these vaccines are administered after initial priming with live vaccines and may require adjuvants to aid in tailoring the immune responses to the immunodominant epitopes [137]. Unfortunately, these adjuvants may as well cause some undesirable reactions in the vaccinated birds. Another disadvantage of the inactivated vaccines is the requirement of a withdrawal period before birds immunised with those vaccines can be processed for human consumption [15]. Additionally, the inactivated ND vaccines are generally poor inducers of mucosal or cell mediated immune response [137]. Thus, to ensure effective protection of chicken against ND, rationally designed vaccines with improved margins of safety and efficacy are required in the poultry industry.

### 4.2. Recombinant Vaccine Technologies 

#### 4.2.1. DNA Vaccines

Advances in recombinant DNA technology have made it possible to develop DNA vaccines by cloning a gene encoding an immunogen or group of neutralising epitopes into an expression plasmid. When the recombinant plasmid is administered into the animal host, the cloned gene can be transcribed and later translated into protein which, when processed by the host cells, could serve as potent epitopes capable of inducing protective immune response [138]. In a recent study, where the complete NDV F gene was cloned in pIRES expression plasmid and used as DNA vaccine in chicken, high antibody titre was observed. In addition, when a plasmid encoding both the F and HN proteins was used as primer to vaccinate chicken and later boosted with inactivated NDV vaccine, a superior protective antibody mediated immunity was observed [139], indicating that these DNA vaccines can be used to improve the effectiveness of inactivated NDV vaccines. The effectiveness of DNA vaccines may be further enhanced when a vehicle such as nanoparticle is used to deliver the vaccine. Firouzamandi et al. [140] used dextran-spermine nanoparticle to encapsulate DNA vaccine encoding NDV F and HN proteins. When the nanoencapsulated vaccine was administered* in ovo*, improvement in HI antibody titre was noticed, although not significantly different from the HI titre obtained from subjects vaccinated with the naked DNA vaccine. Furthermore, immunisation of SPF chicken with a chitosan encapsulated DNA vaccine encoding the NDV F gene leads to an enhanced mucosal and systemic humoral and cell mediated responses [141]. Thus, the DNA vaccine may represent a safe alternative vaccine platform against the current ND challenges. The major selling points of these vaccines are their safety and ability to induce both CD4+ and CD8+ immune responses. However, their limitations include poor immunogenicity, high cost of production, and unsuitability for mass administration. More so, when administered without any delivery vehicle, they are easily degraded by the nucleases before they reach their final destination. Nonetheless, the use of adjuvants and delivery vehicle may overcome some of these limitations [142, 143].

#### 4.2.2. Viral Vector Vaccines

One of the most promising strategies used to combat veterinary and medically important pathogens is the use of recombinant viral vector vaccines. The most common vectors used in poultry are the vaccinia virus, fowl pox virus, and herpes virus of turkeys [144, 145]. Because of their large double stranded DNA genome, vaccinia viruses have a very high capacity of foreign gene expression. They are highly immunogenic, capable of inducing strong inflammatory innate immune response via the TLR activation. Furthermore, they can be easily propagated in large scale using chicken embryo fibroblast cells. Hence, they are frequently used as gene delivery tools against cancer and so many other infectious diseases. Since early 1990s, recombinant vaccinia virus expressing the F gene from NDV strain Italian was shown to induce strong immunity that protected birds against virulent NDV challenge [146]. However, the limitation of this vector such as sensitivity to preexisting immunity against the vector [147] makes its usage in ND vaccine delivery highly limited.

Recombinant fowl pox vectored ND vaccines generated by replacing the thymidine kinase gene with NDV F, HN or F and HN were shown to induce protective immunity against the virulent NDV challenge in chicken [148, 149]. The greatest advantage of the fowl pox vectored vaccine is its inability to cause postvaccinal respiratory reactions in the vaccinated chicken. However, presence of anti-fowl pox virus antibodies in the vaccinated chicken may dramatically interfere with the efficiency of this vector. Furthermore, this vaccine may not be suitable for young birds. Interestingly, this particular shortcoming can be overcome by the use of recombinant herpes virus vectored vaccine which can directly be used in both 18-day-old embryo and 1-day-old chicks [150]. It elicits a strong and long lasting cell mediated and humoral immunity because of its ability to persist as a latent infection in the vaccinated chicken. Furthermore, its gene delivery efficiency is not adversely affected by the presence of preexisting immunity against the backbone vector because it replicates in a kind of cell associated manner [151]. These properties collectively make the vector an ideal vehicle for the delivery NDV immunogens. Indeed recombinant HVT expressing the NDV F glycoprotein was shown to induce 95-100% protection of chicken about 4 weeks after vaccination via the* in ovo* or subcutaneous routes [152]. Thus, vaccination against virulent ND using the HVT vectors remains a promising system for effective disease control in poultry.

Other viral vectors used to deliver NDV vaccines include the avian paramyxovirus-3 (APMV-3). This virus has been shown to replicate excellently in chickens, turkeys, and even cell culture [153]. The virus has also been shown to be highly attenuated in chicken, with ICPI value less than those of most lentogenic NDV isolates [153]. Importantly, preexisting immunity against NDV does not interfere with its efficiency as a vaccine vector. Thus, APMV-3 is an alternative viral vector that can efficiently infect chicken without causing any clinical disease. Recently, Kumar et al. [154] constructed recombinant APMV-3 vaccines vectoring either NDV F or HN. When these vaccines were used to vaccinate 2-week-old chicken, NDV specific cellular and humoral immune responses which protected against virulent NDV challenge were observed. It is however worth mentioning that expressing NDV F or HN in APMV-3 backbone lead to a retardation of replication of the chimeric viruses compared to the wild type APMV-3. Nevertheless, APMV-3 is still regarded as an efficient avirulent vaccine vector in chicken.

#### 4.2.3. Virus Like Particles Platforms

Virus like particles (VLPs) are genome less assemblies of virus structural proteins made up of repetitive surface structures that serve as pathogen associated molecular patterns capable of eliciting a robust immune response [155]. They are morphologically very similar to viruses but are replication incompetent, making them a highly safe vaccine platform [156]. A few years ago, production of NDVLPs was achieved following the expression of M protein in combination with the NP and viral surface glycoproteins (F and HN) [157]. More so, coexpression of NDV F protein and avian influenza M1 protein effectively produced VLPs in baculovirus expression system [158]. Interestingly in the above studies, not only did the VLPs successfully incorporate the surface glycoproteins, but the proteins' structural conformation and biological functions such as fusogenicity, hemagglutination, and neuraminidase activities remained unaffected. Additionally, immunisation of mice or chicken with the VLPs induced strong immune responses similar to those of equivalent amount of inactivated ND vaccines [158, 159]. There are several unique features that differentiate NDVLPs from many other VLP systems. First and foremost, the ratio of the proteins in the VLPs is very similar to that in the wild type virus. Secondly, unlike other VLPs that are released with efficiencies of 10-50%, the NDVLPs were shown to be released with efficiency of 84% from the avian cells [157], making them the VLPs with the highest known release efficiency. Furthermore, the NDVLPs can easily be concentrated and purified to be devoid of any cell content contamination using the established protocols for virus purification. Unfortunately, producing very large amount of VLPs for massive vaccine trial might be challenging especially if platforms other than baculovirus expression systems are used. Furthermore, since VLPs cannot replicate in the vaccinated hosts, they need to be administered individually, in large quantities and with adjuvants in order to achieve effective immune response against the disease [160]. Despite all these challenges, VLPs are still promising safe vaccine platforms that increasingly gain popularity in the control of NDV.

#### 4.2.4. NDV Reverse Genetics-Based Vaccines

The greatest weakness of the conventional genotype II based NDV vaccines is their inability to stop the shedding of the heterologous virulent NDV even if clinical protection is achieved. Although a lot of factors might be involved in this postvaccination shedding of virulent NDV, genotype mis-match between the vaccine and challenge strains is believed to be a key player. Experimentally, it has been shown that virus shedding can substantially be reduced when birds are immunised with vaccines which are homologous to the challenge strains. Hence, the new direction in the fight against ND focuses on the generation of the so-called genotype-matched NDV vaccines.

The latest strategy for the development of genotype-matched live attenuated ND vaccines is reverse genetics, which is the recovery of a recombinant virus from its cloned cDNA [161]. Since the major virulence determinant of NDV has been shown to be the F protein cleavage site [54] whose amino acid composition clearly distinguishes virulent (polybasic) from avirulent (monobasic) strains, reverse genetics can be used to generate genotype-matched ND vaccine, by modifying the cleavage site of the prevalent virulent NDV from polybasic to monobasic [162]. Using this approach, Xiao et al. [163] genetically modified the F cleavage site of a highly virulent NDV circulating in Indonesia and showed that it completely lost its virulence and induces a superb protective immunity that significantly reduced virus shedding following challenge with a highly virulent wild type genotype VII NDV isolate.

In another study, a highly virulent NDV strain JS/5 was used as a back bone to develop a genotype-matched vaccine against genotype VII NDV. By changing the F cleavage site of the virus from polybasic to monobasic, the rescued virus completely lost its virulent phenotype but retained its tropism in chicken embryonated eggs and induced a protective immunity that lead to significant reduction in the shedding of the challenge virus compared to the conventional LaSota vaccine [88]. Therefore, reverse genetics is an attractive technology for rapid generation of stably attenuated genotype-matched vaccines against virulent NDV. Another important application of this technology is in the generation of marker NDV vaccines able to differentiate vaccinated from the infected animals (DIVA). These DIVA vaccines represent an invaluable strategy for sustainable eradication of ND in poultry [162]. At present, reverse genetics-based vaccines are too expensive to develop, because of the high cost of sequencing and other molecular biology services. However, with the increasingly available gene synthesis industries, the cost of these vaccines is likely to drastically reduce in the near future. Therefore, the proliferation of these vaccines in different countries is strongly anticipated in the near future, given their unique characteristics such as protective efficacy, genetic stability, and homogeneity with the prevalent NDV strains.

## 5. Concluding Remarks

To effectively control virulent NDV infection in poultry, it is imperative to rapidly and specifically identify the aetiologic agent. Currently, traditional virus isolation followed by its serological or molecular confirmation is considered to be the gold standard method for NDV detection and pathotyping [2]. However, the lengthy protocol of this assay and its requirement for specialised containment facility diminishes its diagnostic relevance especially under outbreak situations where prompt diagnosis and immediate intervention are required. Molecular based assays such as RT-PCR, qPCR, and LAMP can rapidly detect and differentiate NDV pathotypes based on the chemistry of their F cleavage sites. Unfortunately, NDV virulence is multigenic and other factors independent of the F cleavage site might be important in determining the actual virulence potential of the virus. Thus, the biggest question in the midst of this diagnostic dilemma is ‘what is the best tool for rapid and accurate diagnosis of virulent NDV infection?' Apparently, the answer to this question is NGS, which can specifically and rapidly identify all the virulence markers in the viral genome and simultaneously differentiate mixed infections within a single clinical sample. NGS is, therefore, the most promising emerging technique for the diagnosis and pathotyping of virulent NDV isolates.

An excellent NDV vaccine is that which not only prevents clinical disease, but also reduces or abolishes virus shedding and increases the quantity of the virulent virus required to cause infection [15]. Unfortunately, the currently available inactivated and live attenuated NDV vaccines can only prevent clinical disease but not virus shedding especially following heterologous virus challenge [18]. Yet, they remain in the mainstay of ND control for more than six decades due to their “disease preventing ability” and relatively cheap cost of production. Nevertheless, the search for better alternatives still continues and has so far lead to the emergence of novel vaccine platforms based on recombinant DNA technology. Among these emerging vaccines, the VLPs and DNA vaccines are known for their incredible safety but sadly, they are poorly immunogenic. Recombinant viral vectored NDV vaccines have also demonstrated promising protective efficacy but are significantly affected by the presence of maternally derived antibodies against the vector. So far the most promising vaccines against the virulent NDV infection in poultry are the recombinant genotype-matched live attenuated vaccine candidates generated by reverse genetics. They specifically target the prevailing genotype in a particular region and are therefore rationally designed to fulfil the criteria of an excellent NDV vaccine. It is envisaged that these vaccines will in the near future outshine all the currently available NDV vaccines.
